# Emodin inhibits invasion and migration of hepatocellular carcinoma cells via regulating autophagy-mediated degradation of snail and β-catenin

**DOI:** 10.1186/s12885-022-09684-0

**Published:** 2022-06-18

**Authors:** Binyu Qin, Zhili Zeng, Jianliang Xu, Jing Shangwen, Zeng Jie Ye, Shutang Wang, Yanheng Wu, Gongfeng Peng, Qi Wang, Wenyi Gu, Ying Tang

**Affiliations:** 1 Institute of Tumor, Guangzhou University of Chinese Medicine, Guangzhou, China; 2 Science and Technology Innovation Center, Guangzhou University of Chinese Medicine, Guangzhou, China; 3 Guangzhou University of Chinese Medicine, Guangzhou, China; 4 Hepatobilliary Surgery Department, The Third affiliated Hospital of Su Yat-sen University, Guangzhou, China; 5 Department of Oncology, The First Affiliated Hospital of Guangzhou University of Chinese Medicine, Guangzhou University of Chinese Medicine, Guangzhou, China; 6 Gillion ITM Research Institute, Guangzhou Hongkeyuan, Guangzhou, China; 7 Australian Institute of Bioengineering and Nanotechnology, The University of Queensland, QLD, Brisbane, 4072 Australia

**Keywords:** Emodin, Hepatocellular carcinoma, Autophagy, Epithelial-mesenchymal transition, PI3K/AKT/mTOR, Wnt/β-catenin

## Abstract

**Background:**

Previous studies reported that emodin extracted from *Rheum palmatum L.* exerts antiproliferation and antimetastatic effects in a variety of human cancer types. However, the role of emodin in hepatocellular carcinoma (HCC) remain unknown.

**Methods:**

EdU and colony formation assays were performed to evaluate the effects of emodin on proliferation. The mobility capacities of HCC treated with emodin were evaluated using wound healing assay. Transwell invasion and migration assays were performed to evaluate anti-migratory and anti-invasive effects of emodin on HCC. Annexin V-FITC/PI was performed to analyze the apoptosis. PI stain was performed to analyze cell cycle. RNA sequencing technology was used to identify the differentially expressed genes (DEGs) induced by emodin in HCC. The impact of emodin on autophagic flux in HepG2 cells was examined by mCherry-GFP-LC3 analysis. Western blot was used to assess the protein expressions of epithelial-mesenchymal transition (EMT), autophagy, PI3K/AKT/mTOR and Wnt/β-catenin signaling pathway.

**Results:**

We found that emodin inhibited the growth of HepG2 cells in a dose- and time-dependent manner. In addition, emodin inhibited cell proliferation, induced S and G2/M phases arrest, and promoted apoptosis in HepG2 cells. The migration and invasion of HepG2 cells were also suppressed by emodin. Enrichment analysis revealed that DEGs involved in cell adhesion, cancer metastasis and cell cycle arrest. Moreover, western bolt results show that emodin-induced autophagy promotes Snail and β-catenin degradation. We also found that blocking autophagic flux after emodin treatment caused EMT reversal. Furthermore, the PI3K agonist Y-P 740 significantly reversed the phosphorylation levels of GSK3β and mTOR. These results indicated that emodin induced autophagy and inhibited the EMT in part through suppression of the PI3K/AKT/mTOR and Wnt/β-catenin pathways.

**Conclusion:**

Our study indicated that emodin inhibited cell metastasis in HCC via the crosstalk between autophagy and EMT.

**Supplementary Information:**

The online version contains supplementary material available at 10.1186/s12885-022-09684-0.

## Background

Liver cancer is a frequently occurring cancer around the world, which is primarily isolated into primary liver cancer and metastatic liver cancer. Hepatocellular carcinoma (HCC) is the foremost common sort of liver cancer and is the second leading cause of cancer-related deaths around the world [1]. The highest morbidity of HCC is presented in the Asian countries with China accounting for a huge number of HCC cases [2]. Chronic hepatitis B virus (HBV) infection is the most significant risk factor for HCC in China [3]. Although tremendous progress has been made in HCC treatment, the overall survival of HCC patients remains low and generally results from the late discovery of HCC and lack of effective therapies for advanced and recurred HCC [4]. Sorafenib, a multi-kinase inhibitor, is the only first-line drug approved by FDA for advanced HCC [5]. However, sorafenib treatment only extends the overall survival of patients with advanced HCC by 2-3 months [6]. Epithelial-mesenchymal transition (EMT) is a highly conserved process by which the epithelial cells lose their polarity and become mesenchymal cells. Increasing evidence shows that EMT contributes to tumor metastasis and chemotherapy resistance of liver cancer [7]*.*

**Fig. 1:**
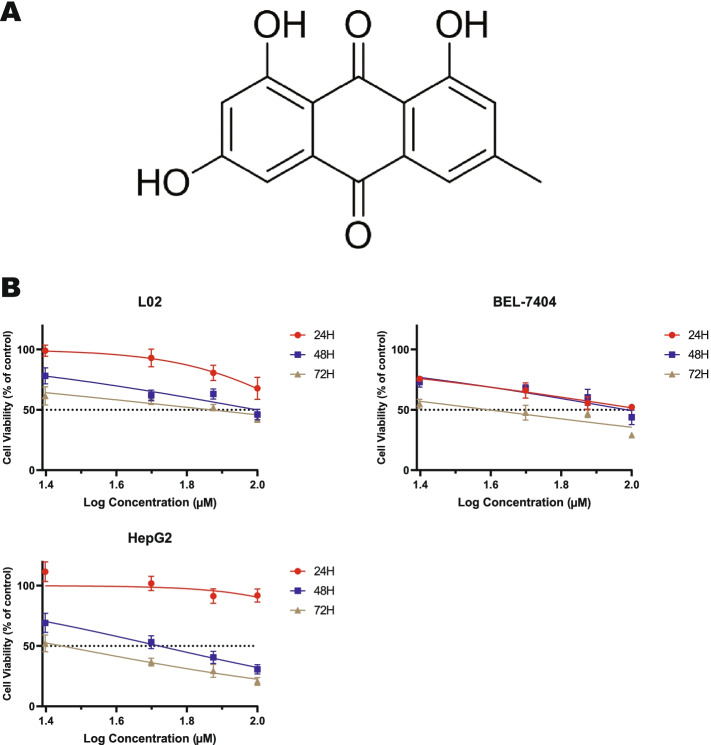
Emodin inhibited cell viability in a dose- and time-dependent manner. **A** Chemical structure of emodin. **B** The effect of emodin on the viability of normal liver cells and HCC was examined by MTT. At least three independent experiments were performed (*n* = 6)

Natural products have been widely used in the development of new pharmaceuticals to treat various diseases, including cancer. Emodin, its structural formula is shown in Fig. 1A, is a kind of natural anthraquinone derivative contained in the traditional Chinese medicines, such as Rheum palmatum, Polygonum cuspidatum and Polygonum multiflorum [8]. In past studies, emodin has been shown to be anticancer [9], liver protection [10], anti-inflammatory [11], and antioxidant [12]. Additionally, several studies have demonstrated that emodin inhibits migration and invasion of cancer cells via EMT [13]. Subramaniam et al. reported that emodin can inhibit cell proliferation and induce apoptosis [14]. However, few studies have investigated the role of emodin in HCC, the molecular target of emodin has not been confirmed. In our previous research, we found that emodin promoted autophagy and apoptosis in HepG2 cells. Autophagy is a protein degradation process in eukaryotic cells, which is closely related to EMT [15]*.* Hence, this research was designed to explore the mechanism of emodin in the proliferation, invasiveness, and migration of HCC.

## Methods

### Chemical compounds and reagents

Emodin was purchased from Yuanye Bio-Technology Co., Ltd. (Shanghai, China; Purity ≥98% by HPLC; lot number: T17O11F127680), which was dissolved in DMSO to provide a stock 50 mM solution and stored at − 20 °C. Fetal bovine serum, culture medium, and other solutions used for cell culture, where specially noted, were from Gibco (Brazil). Chloroquine (CQ), an autophagy inhibitor, was purchased from Selleck Chemicals (TX, USA). PI3K agonist 740 Y-P were purchased from MedChemExpress (Guangzhou, China). Matrigel was purchased from Corning (NY, USA). Dimethyl-sulfoxide was obtained from Sigma-Aldrich Chemical (MO, USA). EdU detection kit and ad-mCherry-GFP-LC3 were purchased from RiboBio Co., Ltd. (Guangzhou, China). Annexin V/PI kit and cell cycle detection kit were purchased from BestBio (Shanghai, China). Trizol was obtained from Invitrogen (CA, USA). The primary antibodies against E-cadherin, N-cadherin, vimentin, PI3K, p-PI3K, AKT and p-AKT were bought from Abcam (Cambridge, MA, USA). The primary antibodies against β-catenin, GSK3β*,* p-GSK3β, Snail, Slug, Beclin1, LC3B, SQSTM1, and p-mTOR were purchased from Cell Signal Biotechnology (Beverly, MA, USA). HRP-conjugated Affinipure Goat Anti-Mouse IgG and Goat Anti-Rabbit IgG were sourced from Proteintech (Chicago, IL, USA).

### Cell line and cell culture

The human liver cancer cell lines HepG2, BEL-7404, BEL-7402, QGY-7701 and Hep3B were obtained from the Chinese Type Culture Collection (CTCC, Shanghai, China). The cells were cultured in DMEM medium with 10% fetal bovine serum (FBS) and 1% penicillin/streptomycin. The Human normal liver cell line L02 (CTCC, Shanghai, China) was maintained in 10% FBS-supplemented DMEM medium and 1% Penicillin/Streptomycin. All the cell lines were incubated under standard conditions at 37 °C in a humidified atmosphere of 5% CO_2_. The cells were passaged twice or thrice a week and discarded after 20 passages. All cells have been tested negative for mycoplasma contamination using Mycoplasma Detection Kit (Beyotime Biotechnology, Guangzhou, China).

### Cell viability assay

Cells were digested and inoculated in 96-well plate at a density of 6000 cells/well, incubated overnight. Afterward, emodin at different concentrations (0, 25, 50, 75, and 100 μM) were used to treat cells for 24, 48, and 72 h. Finally, the cell viability was determined by MTT (Beyotime Biotechnology, Guangzhou, China). Each experiment was repeated three times, and for each experiment 6 replicates were prepared. IC_50_ is defined as the sample concentration at which 50% inhibitions were reached.

### Cell proliferation assay

EdU cell proliferation staining was performed using an EdU kit. Briefly, HepG2 cells were seeded in 24-well plates at 1 × 10^5^ cells/mL and treated with four different concentrations of emodin chosen below its 48 h IC_50_ value, i.e., 0, 15, 30, 60 μM for 48 h. Subsequently, cells were incubated with EdU for 2 h, fixed with 4% paraformaldehyde for 15 min, and permeated with 0.3% Triton X-100 for another 15 min. The cells were incubated with the Click Reaction Mixture for 30 min at room temperature in a dark place and then incubated with Hoechst 33342 for 10 min.

### Cell apoptosis assay

Cell apoptosis staining was performed using the cell apoptosis detection kit according to the manufacturer’s instructions. The cells were washed twice with phosphate-buffered saline (PBS). 1X Binding buffer was added to 1 × 10^6^ cells/mL, and PI was then added in the dark. After incubation for 30 min in the dark, the cells were immediately analyzed using CytoFLEX flow cytometer.

### Cell cycle analysis

At the end of incubation, cells were trypsinized. Cells were collected by centrifugation at 1000 g for 5 min, washed twice with ice-cold PBS, and then fixed with 70% cold ethanol and stored at 4 °C for 24 h. Cells were centrifuged again, washed with cold PBS twice, incubated with RNase A (0.1 mg/mL) for 1 h at 37 °C, and stained with PI (0.1 mg/mL) for 30 min in the dark. The DNA content was measured by flow cytometry, and the percentage of cells in each phase of the cell cycle was evaluated using CytoFLEX flow cytometer and ModFit software.

### Colony formation assay

A density of 400 cells per well seed onto 6-well culture plate and emodin in DMEM medium free of FBS was added to the culture at 3 days after seeding. The culture was continuously maintained for another 14 days and subjected to the colony formation assay. Plates were imaged and colonies were enumerated using Image J (National Institute of Health, USA).

### Wound healing assay

HepG2 cells were seeded in 6-well plates at the density of 3 × 10^5^ cells per well, grown to reach 95% confluency monolayer, and then a “scratch” was made in the cell monolayer using a 100 μL pipette tip to create a wound before drug treatment, afterward the detached cells were washed out using PBS. All wells were treated with different concentrations of emodin. In the same field, the wound distance was measured at 24 h, and 36 h, under DMi1 inverted microscope at 5X magnification (Leica, Germany) to evaluate wound closure. Wound healing was analyzed using Image J. Experiments were carried out in triplicate and repeated at least three times.

### Transwell migration and invasion assays

For the Transwell invasion assay, serum-free medium was applied to dilute the Matrigel (1: 9), and then 50 μl of diluted Matrigel was inoculated into each chamber. After being treated with or without emodin, HepG2 cells were digested and resuspended at a density of 2.5 × 10^5^ cells/mL with DMEM medium without FBS. Then, we added 0.2 mL of the cell suspension to each upper chamber of the 24-well plate, while 0.6 mL DMEM containing 20% FBS was added to the lower chambers. After 24 h, the upper chambers were washed, fixed with 4% paraformaldehyde for 20 min, stained with 0.25% crystal violet for 30 min, and imaged by a microscope. Four fields (10X) were selected randomly, and the number of HepG2 cells was counted. For detection of the migrating ability of cancer cells, the protocol was the same as that for the invasion but without Matrigel.

### Western blot analysis

HepG2 cells were seeded at 1 × 10^6^ cells per T25 flask and incubated overnight. After 48 h of treatment with concentrations of emodin, cells were washed with PBS, and digested with trypsin for 20 min at 4 °C. Cell lysates were centrifuged (12,000 rpm) for 30 min at 4 °C. The protein samples were assessed with BCA Kit, and protein lysates were boiled at 95 °C for 10 min and separated on SDS-PAGE. Then, they were electro-transferred to polyvinylidene fluoride (PVDF) membrane. The membrane was blocked in 5% milk in Tris-buffered saline with 0.1% Tween 20 (TBST) for 1 h at room temperature and washed in TBST for three times. The blots were incubated with primary antibodies overnight at 4 °C. The primary antibodies against E-cadherin, N-cadherin, Vimentin, Snail, Slug, β-catenin, Beclin1, LC3B, SQSTM1, p-AKT, AKT, p-PI3K, PI3K, p-GSK3β, GSK3β, p-mTOR, and β-actin at appropriate dilutions as recommended by the manufacturer. After the membranes were washed in TBST three times, the blots were then incubated with HRP-conjugated Affinipure Goat Anti-Mouse IgG or Goat Anti-Rabbit at room temperature for 1 h. Proteins were visualized by chemiluminescence technology and analyzed with the *Image J*.

### Ad-mCherry-GFP-LC3B transfection

HepG2 cells were seeded on 35 mm diameter confocal dishes until they reached about 70% confluence and then transfected with ad-mCherry-GFP-LC3B adenovirus at an MOI of 20 for 6 h at 37 °C. Following treatment with emodin (60 μM) for 48 h, Finally, images were taken with a confocal microscope (Leica, Germany).

### RNA sequencing

HepG2 cells in 6 different petri dishes were divided into two equal groups. After being treated with emodin for 48 h, total RNA was extracted from treated and untreated cells using Trizol reagent. Agilent 2100 Bioanalyzer (Agilent RNA 6000 Nano Kit) was used to do the total RNA sample QC. Then we assembled those clean reads into Unigenes, followed with Unigene functional annotation, SSR detection and calculate the Unigene expression levels and SNPs of each sample. Finally, we identify differential expressed genes (DEGs) between samples and do clustering analysis and functional annotations. DEGs at each stage or site were used for further analyses of gene ontology (GO) and KEGG pathways using the Database for Annotation, Visualization, and Integrated Discovery (DAVID: http://david.abcc.ncifcrf.gov).

### Gene set enrichment analysis

We explored gene function by Gene set enrichment analysis (GSEA) software Version 2.4.3 (MA, USA) for the down-regulated differential genes. The seven EMT-related gene sets were downloaded from the EMTome web page (EMTome: http://www.emtome.org) [16]. EMT pathways with significant enrichment results were demonstrated on the basis of NES (Net enrichment score) and Nominal *P*-value. Gene sets with |NES| > 1, Nominal *p* < 0.05, and FDR q < 0.25 were considered to be significant [17].

### Statistical analysis

Each experiment was performed at least three times, and all data are presented as the mean ± SD. Statistical analysis was done using GraphPad Prism Version 8.1.0 (CA, USA). One-Way ANOVA tests were used to assess the differences between the means of treated and untreated cells or groups. *P*-values < 0.05 were considered significant (*, *P* < 0.05; **, *P* < 0.010; ***, *P* < 0.001).

## Results

### Emodin inhibits the viability of human liver cancer cells

Emodin, an anthraquinone compound, has been reported to have antitumor effects (Shrimali et al., 2013). To investigate the inhibitory effect of emodin on the growth of liver cancer cells and normal hepatocytes, HepG2, BEL-7404, BEL-7402, QGY-7701, Hep3B and L02 cells were treated with different concentrations of emodin for 24, 48, and 72 h, and monitored by MTT. As showed in Fig. 1B, Supplementary Fig. S1 and Table 1, emodin has inhibited the proliferation of hepatoma cell and normal hepatocytes in a dose and time-dependent manner. Half maximal inhibitory concentration (IC_50_) values were found to be in the range of 73.9 - 133 μM (L02); 52.3 - 208.7 μM (HepG2); 46.7 - 113.5 μM (BEL-7404); 48.0 – 151.3 μM (BEL-7402); 56.9 – 98.4 μM (QGY-7701); 66.1 – 185.4 μM (Hep3B). We selected the HepG2 cell line for our subsequent experiments by the selectivity index (SI) ratios.

**Table 1:** Summary of half-maximal inhibitory concentrations (μM) of emodin on hepatocyte lines

Cell line	L02			HepG2			BEL-7404		
Treatment time	24 h	48 h	72 h	24 h	48 h	72 h	24 h	48 h	72 h
IC_50_	133.05	99.62	73.93	208.71	70.96	52.38	113.57	96.86	46.71
SI^*^				0.63	1.40	1.52	1.17	1.02	1.58
Cell line	QGY-7701			Hep3B			BEL-7402		
Treatment time	24 h	48 h	72 h	24 h	48 h	72 h	24 h	48 h	72 h
IC_50_	98.46	71.03	56.99	185.40	115.37	66.11	151.30	84.81	48.09
SI^*^	1.35	1.40	1.29	0.71	0.86	1.11	0.87	1.17	1.53

*SI Selectivity index**,** IC_50_ value against normal liver cells/IC_50_ value against HCC

### Emodin inhibits HepG2 cells proliferation, clonal formation ability and cell cycle progression

**Fig. 2:**
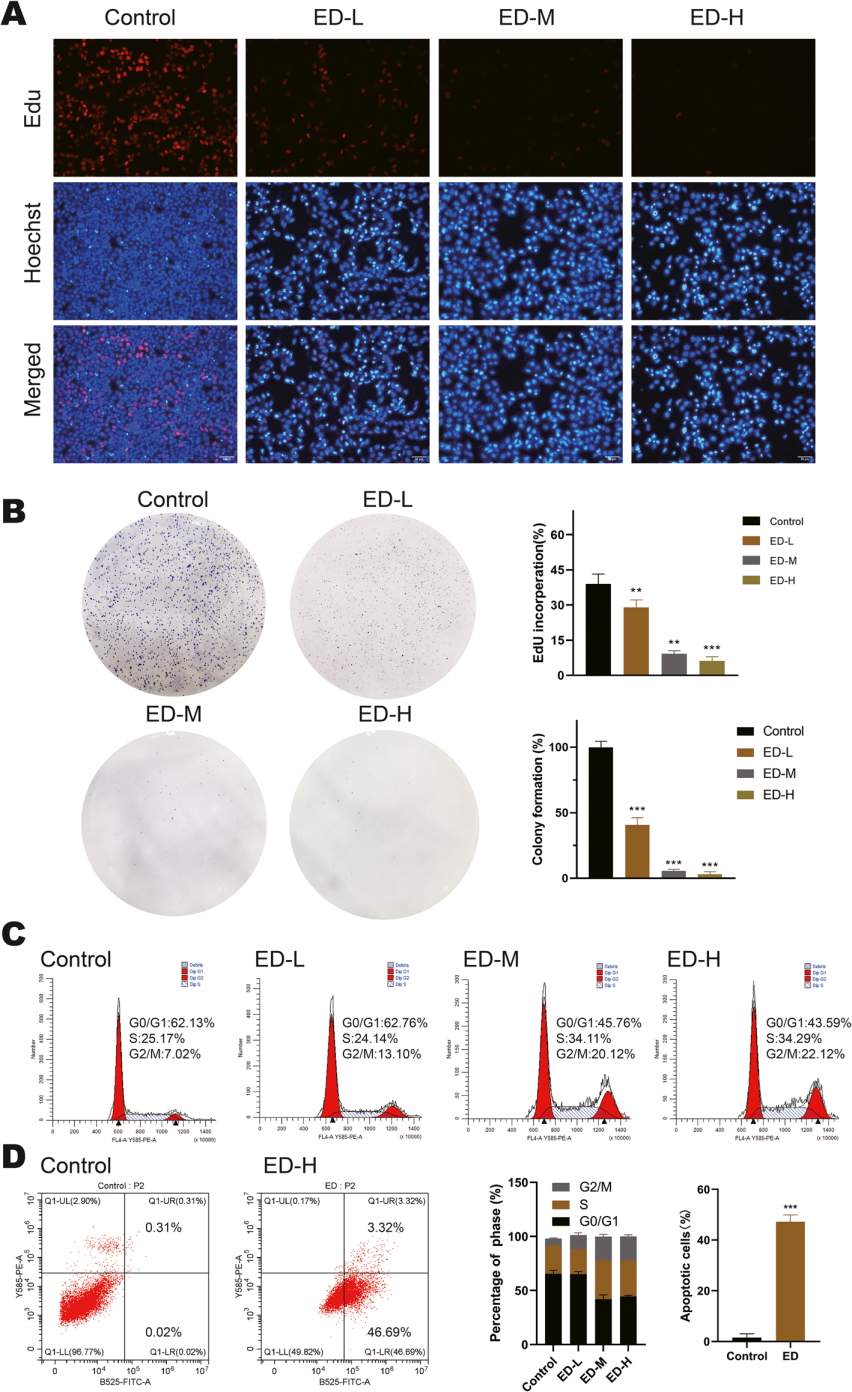
Effects of emodin on cell proliferation, apoptosis and cell cycle of HepG2 cells. **A** The number of EdU-positive cell rate was detected by EdU staining after 48 h of treatment. (Scale bar, 50 μm). The percentage of cells with EdU incorporation was quantified by Image J. EdU incorporation = cells stained by red/cells stained by blue × 100%. **B** Colony formation assay of cells treated with various concentrations of emodin for 48 h, the number of colonies was assessed after two weeks.  **C** Flow cytometry analysis of the cell cycle following treatment with various concentrations of emodin for 48 h and the quantification of the cell cycle distribution was performed by ModFit software. **D** After emodin (60 μM) treatment of cells for 48 h, apoptosis was determined by flow cytometry with Annexin V/PI double staining. Each experiment was repeated at least three times; representative images are shown. Data were expressed as mean ± SD. * *P* < 0.05. ED-L: emodin (15 μM), ED-M: emodin (30 μM), ED-H: emodin (60 μM)

To further investigate the effect of the proliferation of HepG2 cells at various concentrations of emodin, we performed EdU staining assay. The results showed that the high-dose group significantly inhibited HepG2 cells proliferation compared with the control group in Fig. 2A. Moreover, the colony formation assay was performed to investigate the ability of HepG2 cells to form colonies. The results were shown in Fig. 2B*,* emodin concentration-dependently suppressed colony formation compared with control group. The effects of emodin were also identified on the cell cycle and apoptosis of HepG2 cells using flow cytometry (Fig. 2C, D). It was found that emodin inhibited cell proliferation in a dose dependent manner after 48 h of treatment and induced cell apoptosis. The percentage of cells in S and G2/M phases was dramatically increased compared with the control group, whereas the proportion of G0/G1 phase decreased. Annexin V/PI staining showed that the apoptotic HepG2 cell percent increased to 50.01% compared to 0.34% at 60 μM concentration of emodin. In summary, our results suggest that emodin plays an important role in the control of HepG2 cell growth, colony formation and cell cycle regulation.

### Emodin inhibits migration and invasion in HepG2 cells

**Fig. 3:**
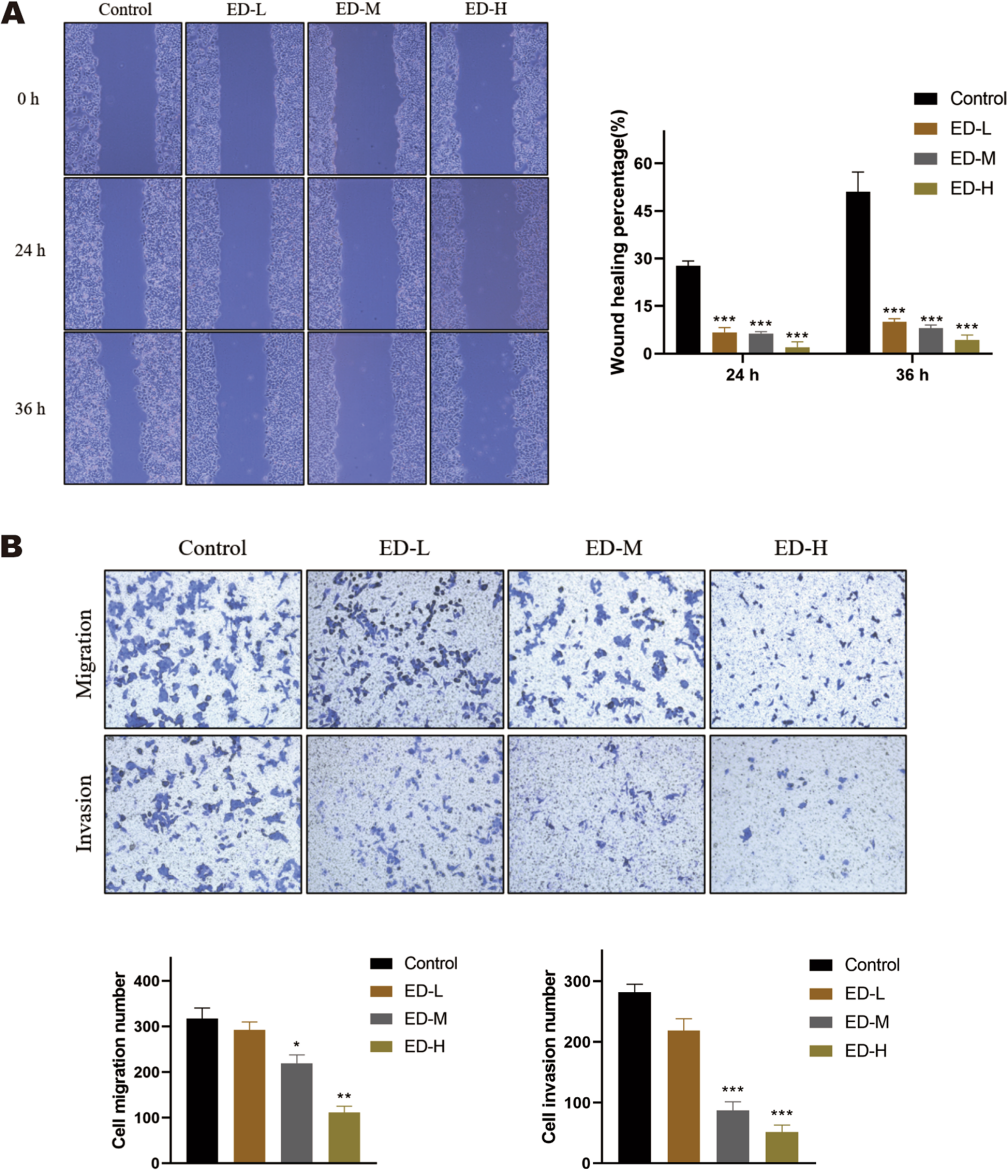
Effects of emodin on invasion and migration potential of HepG2 cells. **A** The scratch assay was used to investigate the effect of emodin on the wound healing ability at 0, 24, and 36 h. The wound healing rate was calculated as (the width of initial wound - the width of remaining wound) / the width of initial wound. (magnification, × 50). **B** Cells were treated with various concentrations of emodin for 48 h, transwell migration and transwell invasion assays were performed on treated cells. (magnification, × 100). Each experiment was repeated at least three times; representative images were shown. Data were expressed as mean ± SD. * *P* < 0.05

Migration capability was detected by the scratch wound healing, transwell migration and invasion assays. The results revealed that gap widths were obviously suppressed after treatment with emodin (15 μM, 30 μM, and 60 μM) in 24 h and 36 h compared with untreated cells (Fig. 3A). Additionally, transwell migration assays also demonstrated that the migration ability of HepG2 cells was also markedly inhibited compared with control cells following treatment with emodin (15 μM, 30 μM, and 60 μM) at 24 h (Fig. 3B). Finally, we investigated the effects of emodin on cell invasion using matrigel-coated transwell invasion assays. As shown in Fig. 3B, emodin significantly reduced the invasion of HepG2 cells at 30 μM and 60 μM compared with control group at 24 h. The results suggested that emodin could decrease migration and invasion of HepG2 cells in a dose dependent manner.

### Gene set enrichment analysis of HepG2 cells upon treatment with emodin

**Fig. 4:**
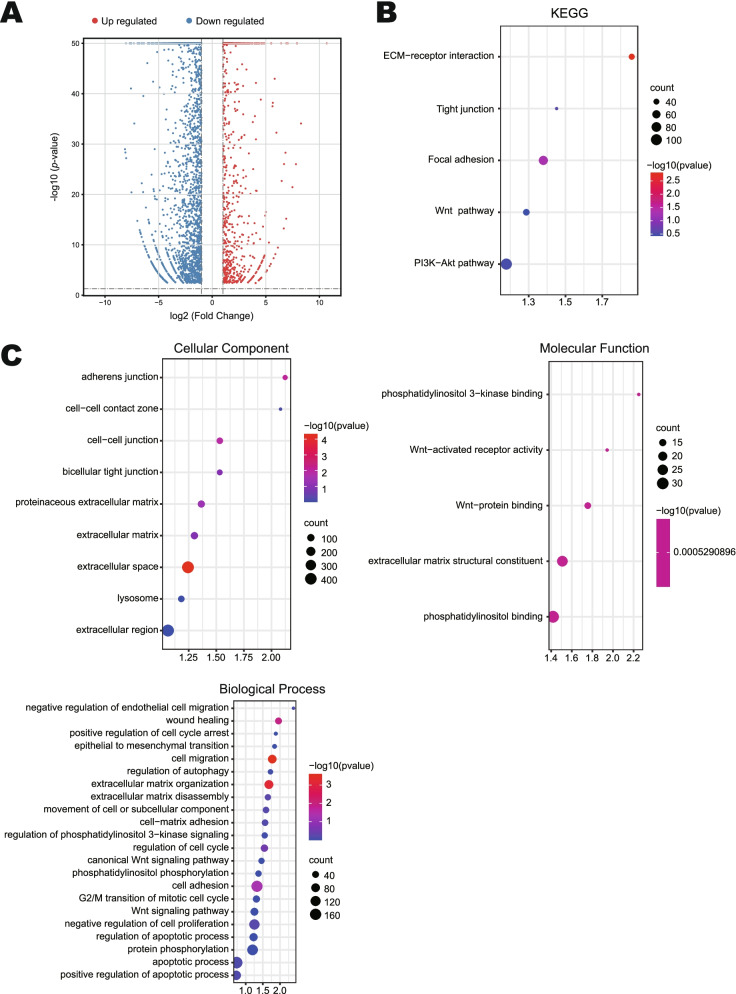
Enrichment analysis of DEGs related to invasion and metastasis in HepG2 cells treated with emdin. **A** Volcano plot of DEGs. Normalized expression data expressed as log2 values, grouped by normal (*n* = 3) and emodin (*n* = 3). **B** KEGG enrichment results of DEGs. **C** CC, MF and BP enrichment results of DEGs. *P* < 0.05. DEGs: differentially expressed genes

DEGs in HepG2 cells were identified by using RNA-seq after treatment with 60 μM emodin at 48 h. As shown in Fig. 4A, sequencing revealed 4989 DEGs comprising 1284 genes that were upregulated and 3705 genes that were down-regulated (*P* < 0.05, |log2 (fold change)| > 1)*.* To further understand the biological classifications of DEGs, we conducted GO and KEGG enrichment analysis using DAVID. The results of KEGG and GO enrichment analysis related to cell cycle, invasion, and metastasis are as follows. Changes in cell component (CC) of DEGs were signifcantly enriched in adherens junction, cell-cell contact zone, cell-cell junction, bicellular tight junction, proteinaceous extracellular matrix, extracellular matrix, extracellular space, lysosome, and extracellular region. Changes in molecular function (MF) of DEGs were mainly enriched in Wnt-activated receptor activity, phosphatidylinositol 3-kinase binding, Wnt-protein binding, extracellular matrix structural constituent, and phosphatidylinositol binding. Changes in biological processes (BP) of DEGs were mainly enriched in negative regulation of cell proliferation, epithelial to mesenchymal transition, cell migration, extracellular matrix organization, wound healing, cell adhesion, regulation of cell cycle, extracellular matrix disassembly, movement of cell or subcellular component, cell-matrix adhesion, positive regulation of apoptotic process, apoptotic process, negative regulation of endothelial cell migration, regulation of autophagy, protein phosphorylation, epithelial to mesenchymal transition, canonical Wnt signaling pathway, G2/M transition of mitotic cell cycle, positive regulation of cell cycle arrest, Wnt signaling pathway, phosphatidylinositol phosphorylation, regulation of apoptotic process (Fig. 4C). In the GO analysis, most of the biology processes were related the cell adhesion, EMT and migration. After KEGG analysis, DEGs were mainly enriched in ECM-receptor interaction, Focal adhesion, Wnt signaling pathway, Tight junction, and PI3K/AKT signaling pathway (Fig. 4B). Functional enrichment analysis of the DEGs revealed that the PI3K/AKT pathway and Wnt signaling pathway were significantly enriched.

### Emodin prevented HepG2 cells EMT

**Fig. 5:**
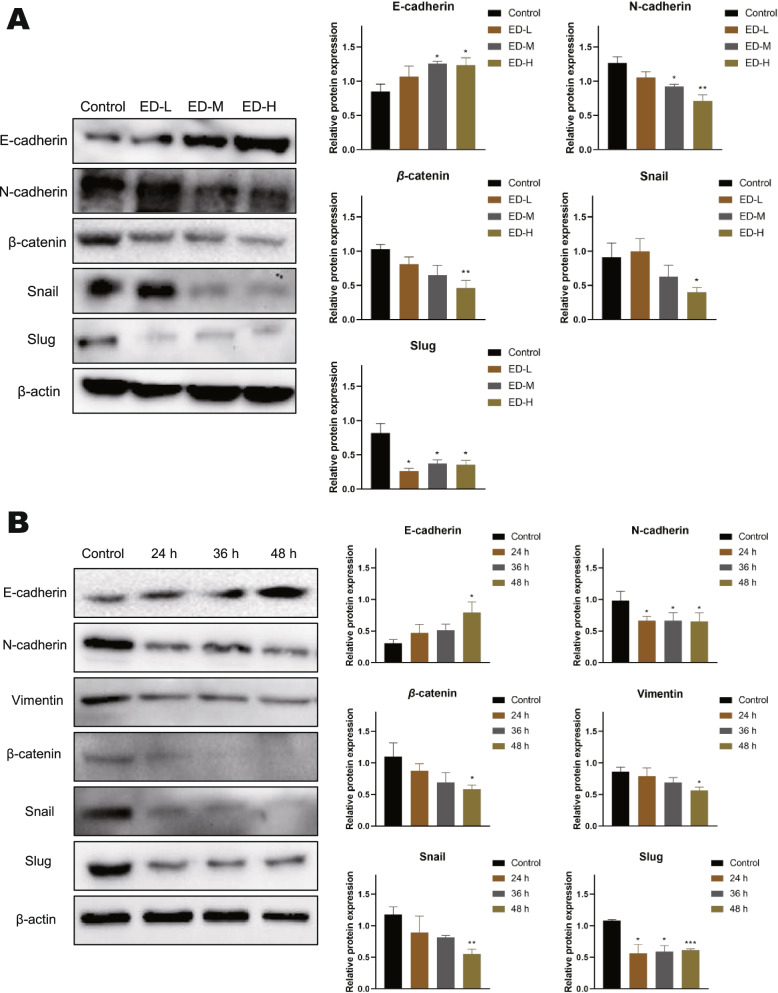
Effects of emodin on EMT-related proteins expression of HepG2 cells. **A** Cells were treated with various concentrations of emodin for 48 h, and then the expression levels of E-cadherin, N-cadherin, β-catenin, Snail, and Slug were tested by western blot. **B** Cells were treated with 60 μM emodin for 24, 36, and 48 h, and then the expression levels of E-cadherin, N-cadherin, Vimentin, β-catenin, Snail, and Slug were tested by western blot. β-actin was loaded as controls. Each experiment was repeated at least three times. Data were expressed as mean ± SD. * *P* < 0.05

Because EMT process has been strongly associated with migration and invasion in cancers. Besides, The GSEA analysis based on seven gene sets consistently showed that the EMT pathway was significantly enriched in down-regulated genes, with the |NES| ranging from 1.587 to 1.983 (all *P* value < 0.05). (supplementary Fig. S1), and therefore, the expression of E-cadherin, N-cadherin, β-catenin, vimentin, Snail, and slug was examined by western blot. As is shown in Fig. 5A, the protein expression of E-cadherin in HepG2 cells was significantly upregulated at 30 μM and 60 μM for 48 h. The protein expression of N-cadherin, β-catenin, Snail, and Slug were dramatically downregulated in a dose-dependent manner. Moreover, we examined the protein expression of EMT at different times. As is shown in Fig. 5B, high concentrations of emodin (60 μM) treatment enhanced the protein expression of E-cadherin but decreased N-cadherin, β-catenin, vimentin, Snail, and slug. Therefore, emodin partly reversed the EMT in a dose-dependent manner in HepG2 cells.

### Emodin inhibits HepG2 cells EMT is associated with autophagy

**Fig. 6:**
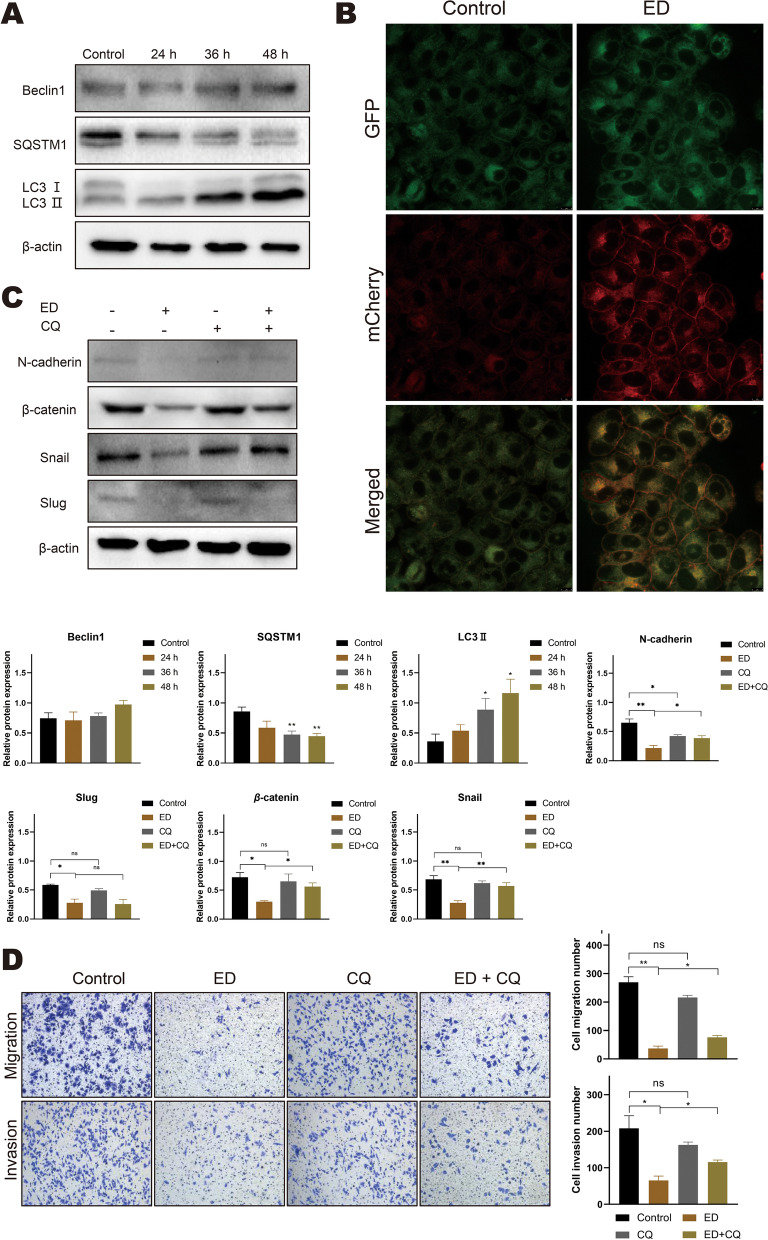
Emodin can regulate autophagy in HepG2 cells. The combination of emodin and CQ reversed the EMT process. **A** Cells were treated with 60 μM emodin for 24, 36, and 48 h, and then the expression levels of Beclin1, p62/SQSTM1 and LC3 II were detected by western blotting. **B** Confocal images of mCherry-GFP-LC3 cells after treatment with 60 μM emodin for 48 h. (Scale bar, 8 μm). **C** Cells were treated with or without emodin (60 μM) in the absence or presence of CQ (10 μM) for 48 h, and then the expression levels of N-cadherin, β*-*catenin, Snail and Slug were detected by western blot analysis. β-actin was loaded as controls. **D** Cells were treated with or without emodin (60 μM) in the absence or presence of CQ (10 μM) for 48 h, Transwell migration and transwell invasion assays were performed on treated cells. (magnification, × 100). Each experiment was repeated at least three times; representative images are shown. Data are expressed as mean ± SD. * *P* < 0.05. CQ: chloroquine, ns: non-significant

As is well known, autophagy and EMT play an important role in cancer progression. Some studies indicated that autophagy promotes metastasis and survival of cancer cells, but on the other hand, autophagy can suppress the activation of EMT. To further explore the mechanism of emodin-regulated EMT in HepG2 cells, we validated whether emodin influences autophagy flux by western blot and immunofluorescence. As is shown in Fig. 6A, B, the LC3 II/β-actin ratio was significantly upregulated with emodin treatment of 60 μM for 24, 36, and 48 h. In addition, emodin notably decreased the expression of SQSTM1, however, there was no significant difference in Beclin1 expression compared with control group. Consistent with the above observations (Fig. 6B), the immunofluorescence results indicated that emodin increases the accumulation of yellow LC3 puncta. To further verify the function of autophagy on EMT, the autophagy inhibitor (CQ) pretreatment followed by 60 μM emodin stimulation for 48 h. As is shown in Fig. 6C, the protein expression of N-cadherin, β-catenin, and Snail could be reversed by co-treatment compared with emodin alone. However, no significant changes in Slug levels. Similarly, the transwell assay results showed that combination treatment with emodin and CQ reverses the inhibitory effects of emodin on cell migration and invasion (Fig. 6D).

### The regulatory effects of emodin on autophagy and EMT are partially attributed to the PI3K/AKT signaling and Wnt/β-catenin signaling

**Fig. 7:**
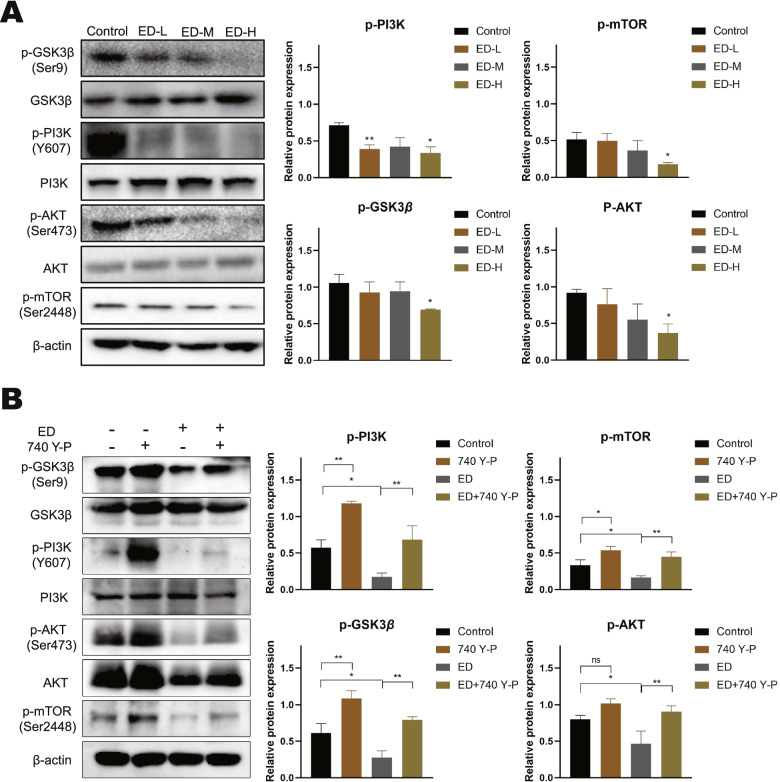
Effect of emodin on PI3K/AKT/mTOR and Wnt/β-catenin pathways. **A** Cells were treated with various concentrations of emodin for 48 h, and then the expression levels of p-GSK3β (Ser9), GSK3β, p-PI3K (Y607), PI3K, p-AKT (Ser473), AKT, and p-mTOR (Ser2448) were detected by western blotting. **B** Cells were pretreated with 15 μM 740 Y-P for 24 h, followed by treatment with 60 μM emodin for 48 h. β-actin was loaded as controls. Each experiment was repeated at least three times. Data are expressed as mean ± SD. * *P* < 0.05

Based on the above results, we hypothesized that the PI3K/AKT/mTOR and Wnt/β-catenin signaling pathways are involved in emodin-mediated inhibition of EMT in HepG2 cells. We have evaluated the expressions of PI3K, p-PI3K, AKT, p-AKT, p-mTOR, GSK3β, and p-GSK3β by western blotting (Fig. 7A). The results indicated that no significant effects in expression of PI3K, AKT, and GSK3β after 48 h treatment with various doses of emodin. While, emodin reduced the expression of all the phosphorylated proteins in a concentration-dependent manner in HepG2 cells. It is well known that PI3K/AKT/mTOR signaling pathway is an intracellular signaling pathway that negatively regulates autophagy. mTOR is an important regulator of autophagy, and phosphorylation of AKT is critical to the activation of mTOR [18]. Moreover, growing evidences suggest that Wnt/β-catenin pathway is not only the classical pathway to regulate the EMT-related genes in malignant tumors, but also has a complex association with autophagy at different stages. GSK3β is the critical downstream regulator of the Wnt pathway and has been shown to induce autophagy [19]. Interestingly, in our study, emodin downregulated the phosphorylation of GSK3β (Ser9), itself a direct target of AKT [20]. To further verify the effect of emodin on PI3K/Akt signaling, the agonist of PI3K/Akt signaling 740 Y-P was used to treat the HepG2 cells. After pretreatment with 740 Y-P for 24 h, HepG2 cells were treated with 60 μM emodin for 48 h. As is shown in Fig. 7B, agonist 740 Y-P reversed the inhibitory effects of emodin in HepG2 cells.

## Discussion

HCC as an aggressive disease is still a major clinical problem due to the lack of effective early diagnosis and treatment. The compounds of natural plants or Chinese herbal medicines have shown some benefits in the prevention and treatment of HCC [21–23]. Emodin exhibits anticarcinogenic activity among various tumors, especially in HCC. In HCC, emodin plays a therapeutic role by inhibiting the growth and migration and inducing apoptosis and cell cycle arrest [24]. For instance, emodin induced cell apoptosis through PI3K/AKT signaling pathways in HCC [25]. Furthermore, emodin succinyl ester was reported to inhibits the proliferation and migration of HCC by targeting the interaction of AR and EZH2 [26]. In addition, emodin has been shown that it can effectively improve the sensitivity of cancer cells to drug therapy, hence manifesting a synergistic anti-cancer effect. Sun et al. have reported that co-administration of aloin and metformin notably suppress growth and invasion, as well as advance apoptosis and autophagy in HCC [27]. However, to our knowledge, none have investigated the underlying mechanism between emodin-induced liver cancer cell autophagy and EMT. In this work, we found that emodin induced autophagy via the PI3K/AKT/mTOR pathway, which inhibited Wnt/β-catenin pathway activity and facilitated transcription factor Snail clearance to modulate EMT in HCC. so we hypothesized that emodin might prevent the EMT by effecting the crosstalk of autophagy and Wnt/β-catenin signaling in HCC.

HCC is characterized by avoidance of apoptosis and highly proliferative [28]. Inhibition of cancer cells growth through cell cycle arrest is one of the main mechanisms whereby anti-cancer drugs function. It is meaningful to find more effective drugs to treat HCC. Emodin plays an important role in inducing cell cycle arrest in a variety of cancer cells. Cheng et al. reported that aloe-emodin can strengthen the sensitivity of adriamycin to breast cancer cell by inhibiting G2/M phase, inducing apoptosis through DNA damage, ROS accumulation, and caspase-3 activation [29]. Dong et al. found that emodin exerts inhibitory effects on the proliferation of HCC cells through restraint of S and G2/M phases [30]. Interestingly, the study showed that emodin can inhibit G0/G1 and G2/M phase arrest in human gastric cancer cells MKN45, and the potential mechanisms of emodin and aloe-emodin induced cell death were found to be different by analyzing intracellular polyamine levels and DNA fragmentation [31]. Our study shows that emodin inhibited cell proliferation, induced S and G2/M phases arrest, and triggered apoptosis in HepG2 cells, it is consistent with results reported previously. Previous studies have confirmed that PI3K/AKT signaling pathway is critical for the cell proliferation, cell cycle arrest, and apoptosis of HCC [32]. In the present study, we demonstrated that emodin inhibited the phosphorylation of the PI3K/AKT signaling pathway in HepG2 cells (Fig. 7). In addition, our results revealed that emodin activated autophagy and upregulated autophagic flux in a time-dependent manner. The interaction between autophagy and apoptosis is complex and is involved in the regulation of the cell cycle [33, 34], and the underlying molecular mechanisms need further study. Collectively, emodin played the antitumor effect by inhibiting cell proliferation, inducing cell cycle arrest, and apoptosis in HCC through PI3K/AKT pathway.

Metastasis is the leading cause of death in patients with HCC [35]. HCC patients with distant metastases are almost lost to surgical treatment, and most systemic therapies have proven unsuitable for treating patients with HCC [36]. EMT plays an important role in the invasion and metastasis of cancer cells. It is closely related to the progression and prognosis of HCC [37]. Following the treatment with emodin in HepG2 cells, migration and invasion of cells were notably suppressed (Fig. 3), EMT-related markers, such as N-cadherin, Snail and Slug, were down-regulated (Fig. 5). In addition, E-cadherin expression was observed to be upregulated and it has an important role in maintaining epithelial cell polarity and function. Within the EMT-associated transcription factors, the Snail family including of Snail and Slug are the most widely studied and overexpressed in HCC [38], which can control migration by directly influencing the synthesis of E-cadherin and N-cadherin [39]. Wnt/β-catenin pathway is one of the key regulatory pathways of EMT. In HCC, β-catenin expression is upregulated and often mutated, which modulates transcription of Wnt target genes [40]. During activation of the canonical Wnt signaling, β-catenin accumulated in the cytoplasm enters the nucleus to interact with T-cell factor/ lymphoid enhancer factor (TCF/LEF), which directly regulates EMT-related genes such as Snail [41]. Furthermore, GSK3β is also a key protein in canonical Wnt signaling, which is a negative regulator of Wnt signaling pathway and a major component of the β-catenin destruction complex. AKT can phosphorylate GSK3β Ser9, thereby allowing β-catenin to escape degradation [42]. We showed for the first time that emodin significantly inhibited the protein expression of β-catenin and the phosphorylation of GSK3β in HCC, thus suggesting that emodin may suppressed Wnt/β-catenin activity (Fig. 5, and Fig. 7). The transcriptomic analysis that we conducted further confirmed this (Fig. 4). However, whether emodin inhibits the nuclear translocation of β-catenin remains unknown, which requires further validation.

Autophagy is an intracellular lysosomal pathway that is involved in protein degradation. mTOR is an important downstream regulator of the PI3K/AKT pathway and is associated with autophagy [43]. Previous studies have confirmed that many factors inhibit HCC by inactivating the PI3K/AKT/mTOR signaling pathway [43]. In addition, autophagy has been suggested to play a paradoxical role in the metastasis of cancer. On the one hand, in Most tumors, hypoxic tumor microenvironment often causes EMT, while the EMT process requires autophagy to ensure cancer cell survival. Previously, it was reported that the protein level of Atg5 (autophagy-related 5) demonstrates positively correlated to the invasiveness of human pancreatic cancer [44]. Furthermore, they found that knockdown of Atg5 significantly inhibits tumor growth and metastasis in vitro. Kudo Y et al. reported that loss of protein kinase C promotes autophagy and facilitates liver cancer metastasis [45]. On the other hand, a growing number of studies demonstrate that autophagy can reverse EMT. It has been shown that inhibition of autophagy promotes SQSTM1-mediated NF-κB activation and increases the expression of EMT-related transcription factors in Ras-mutated cancer cells [46]. SQSTM1 is an autophagy marker and negatively correlates with autophagic flux. The authors also reported that FAT4 promotes autophagy through PI3K/AKT signaling pathway to inhibit invasion and migration of colorectal cancer [47]. In our results, the PI3K/AKT/mTOR signaling pathway was inhibited, emodin decreased the phosphorylation levels of PI3K, AKT, and mTOR. In addition, LC3II accumulation, SQSTM1 reduction, and formation of LC3 punctae indicated that emodin significantly induced autophagic activity (Fig. 6). Overall, emodin played an important role in regulating autophagy through targeting PI3K/AKT/mTOR pathway to achieve antitumor activity.

**Fig. 8:**
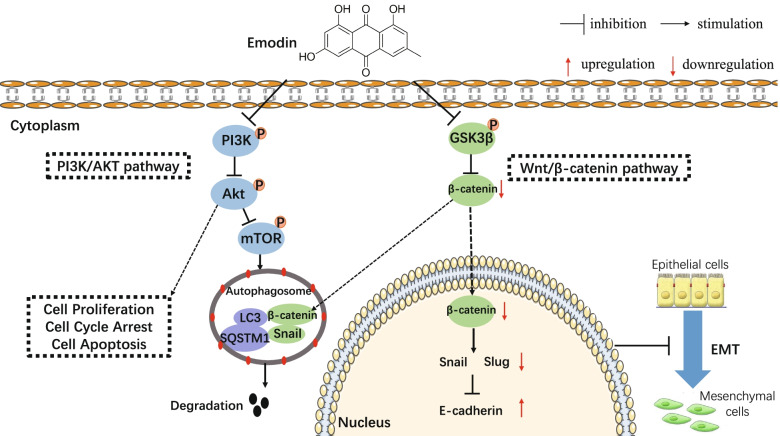
Schematic illustration of emodin effects in the crosstalk between autophagy and EMT in HepG2 cells

Our studies have shown that emodin induced autophagy and inhibited EMT in HepG2 cells. To explore the exact function of autophagy in EMT markers, we used CQ to inhibit the autophagic flux. As expected, increased epithelial marker expression and decreased mesenchymal marker expression were observed in HepG2 cells in the use of emodin alone. In contrast, decreased the expression of epithelial markers and increased the expression of mesenchymal markers were observed when combined with CQ. Emodin combined with CQ significantly down-regulated the proteins expression of Slug but not Snail. These results suggest that autophagy-dependent degradation of Snail is associated with EMT-related proteins. Moreover, other studies have also reported that autophagy inhibits EMT and tumor metastasis through degradation of Snail [48, 49]. In addition to Snail, emodin-induced autophagy also promoted β-catenin degradation in HepG2 cells. Snail and β-catenin are selectively degraded by combining with LC3 through LC3 interaction area (LIR) [50, 51]. Collectively, emodin may inhibit EMT by promoting the degradation of β-catenin, snail via autophagy, blocking Wnt signalling in HepG2 cells. The mechanism underlying of autophagy and EMT is complicated, and Wnt/β-catenin pathway may play a significant role in the crosstalk between autophagy and EMT (Fig. 8). However, due to the limitations of the study conditions, this study was not validated in a suitable animal model, the detail mechanism should be elucidated further.

## Conclusion

In summary, this study showed that emodin inhibited cell proliferation, induced S and G2/M phases arrest, and triggered apoptosis through PI3K/AKT signaling pathway in HCC. And emodin inhibited EMT through the crosstalk between autophagy and Wnt/β-catenin. In conclusion, our findings further elucidated the potential mechanism of emodin in HCC cells metastasis and confirmed the anticancer effect of the compounds from Traditional Chinese Medicine, which deserves further exploration to obtain more valuable drugs for the treatment of HCC.

## Supplementary Information

**Additional file 1.**

**Additional file 2.**

**Additional file 3.**

**Additional file 4.**

**Additional file 5.**

## Data Availability

All data generated or analysed during this study are included in this published article.

## Abbreviations

HCC: Hepatocellular Carcinoma
HBV: Hepatitis B Virus
EMT: Epithelial-mesenchymal transition
CQ: Chloroquine
FBS: Fetal bovine serum
PBS: Phosphate-buffered Saline
TBST: Tris-buffered saline with Tween 20
DMSO: Dimethylsulfoxide
DEGs: Differentially expressed genes
SI: Selectivity index
IC_50_: Inhibitory concentrations of 50%
BP: Biological processes
CC: Cell component
MF: Molecular function
GO: Gene ontology
KEGG: Kyoto Encyclopedia of Genes and Genomes
