# Supplementary material for: Emodin inhibits invasion and migration of hepatocellular carcinoma cells via regulating autophagy-mediated degradation of snail and β-catenin
Source: BMC Cancer. 2022 Jun 18;22:671. doi: 10.1186/s12885-022-09684-0 (PMC9206273; doi:10.1186/s12885-022-09684-0)
Supplement: Supplementary file 1 — Additional file 1. [file 12885_2022_9684_MOESM1_ESM.pdf]

Figure S 5A

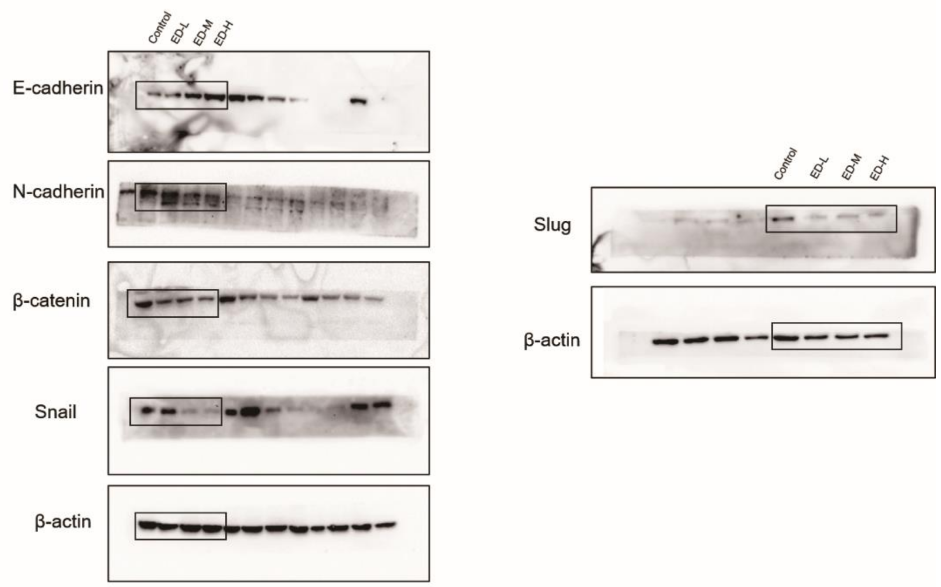

Figure S 5B

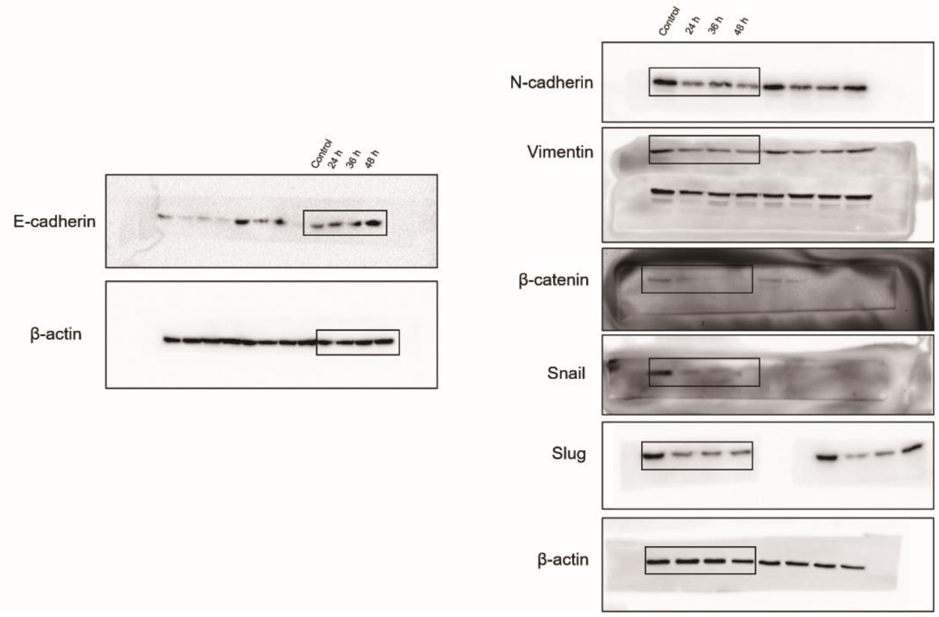

Fig. S5. The original gel pictures of Fig. 5. (A) Cells were treated with various concentrations of emodin for 48 h, and then the expression levels of E-cadherin, N-cadherin, β-catenin, Snail, and Slug were tested by western blot. (B) Cells were treated with 60 μM emodin for 24, 36, and 48 h, and then the expression levels of E-cadherin, N-cadherin, Vimentin, β-catenin, Snail, and Slug were tested by western blot. The bands framed by the black line have been cropped by the original whole gel images.
