# Supplementary material for: Emodin inhibits invasion and migration of hepatocellular carcinoma cells via regulating autophagy-mediated degradation of snail and β-catenin
Source: BMC Cancer. 2022 Jun 18;22:671. doi: 10.1186/s12885-022-09684-0 (PMC9206273; doi:10.1186/s12885-022-09684-0)
Supplement: Supplementary file 2 — Additional file 2. [file 12885_2022_9684_MOESM2_ESM.pdf]

Figure S 6A

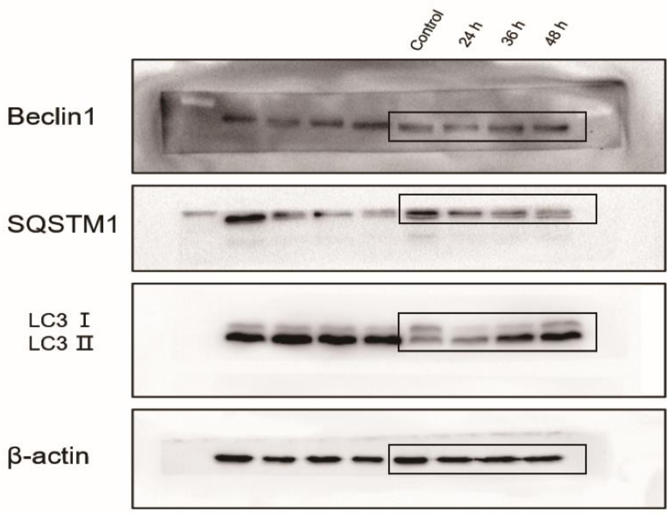

Figure S 6C

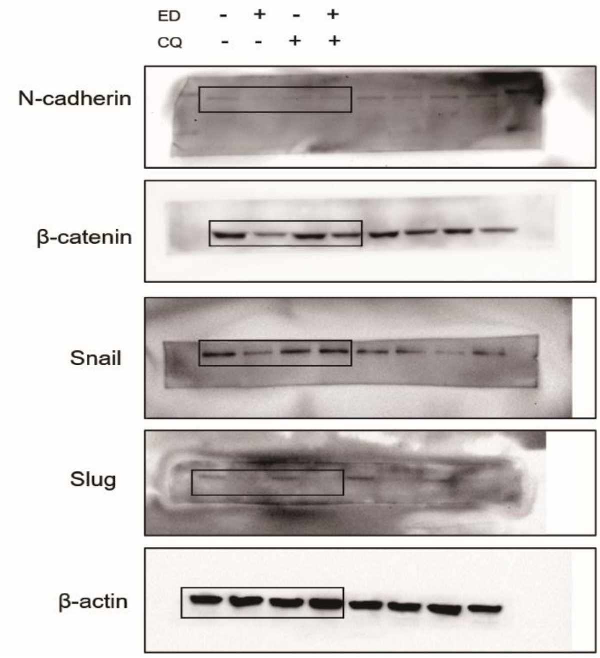

Fig. S6. The original gel pictures of Fig. 6. (A) Cells were treated with 60 μM emodin for 24, 36, and 48 h, and then the expression levels of Beclin1, P62 and LC3 II were detected by western blotting. (C) Cells were treated with or without emodin (60 μM) in the absence or presence of CQ (10 μM) for 48 h, and then the expression levels of N-cadherin, β-catenin, Snail and Slug were detected by western blot analysis. β-actin was loaded as controls. The bands framed by the black line have been cropped by the original whole gel images.
