# Supplementary material for: Emodin inhibits invasion and migration of hepatocellular carcinoma cells via regulating autophagy-mediated degradation of snail and β-catenin
Source: BMC Cancer. 2022 Jun 18;22:671. doi: 10.1186/s12885-022-09684-0 (PMC9206273; doi:10.1186/s12885-022-09684-0)
Supplement: Supplementary file 3 — Additional file 3. [file 12885_2022_9684_MOESM3_ESM.pdf]

Figure S 7A

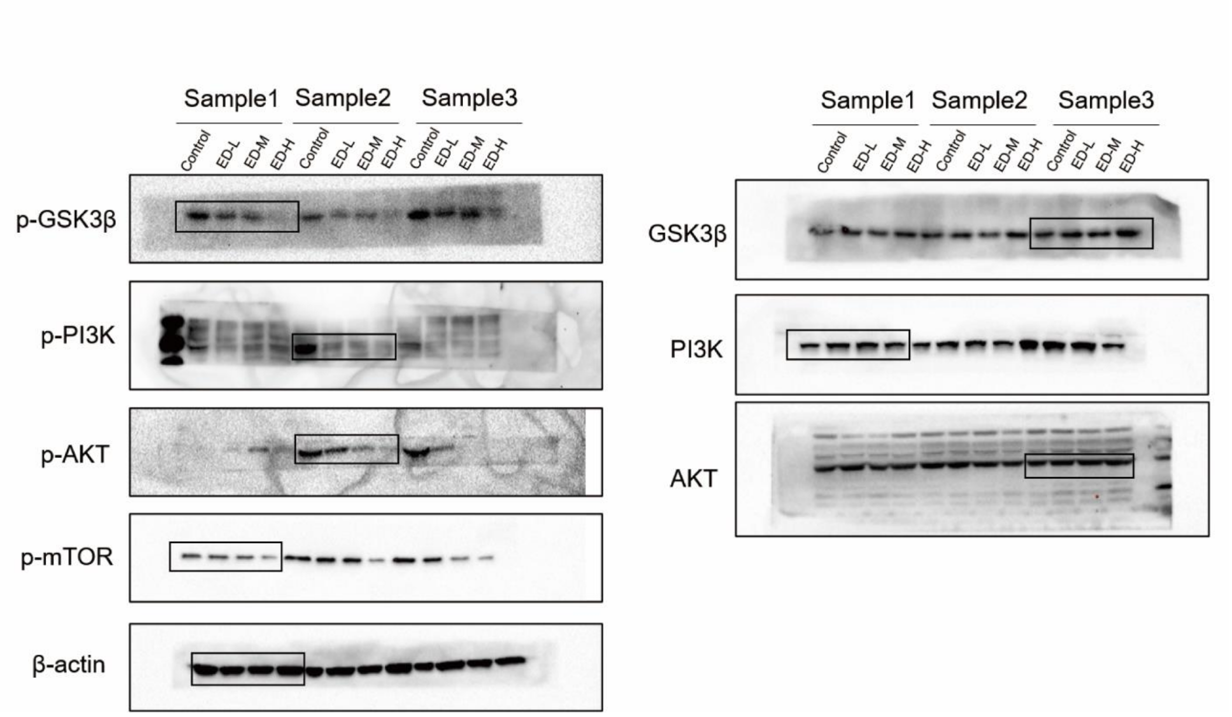

Figure S 7B

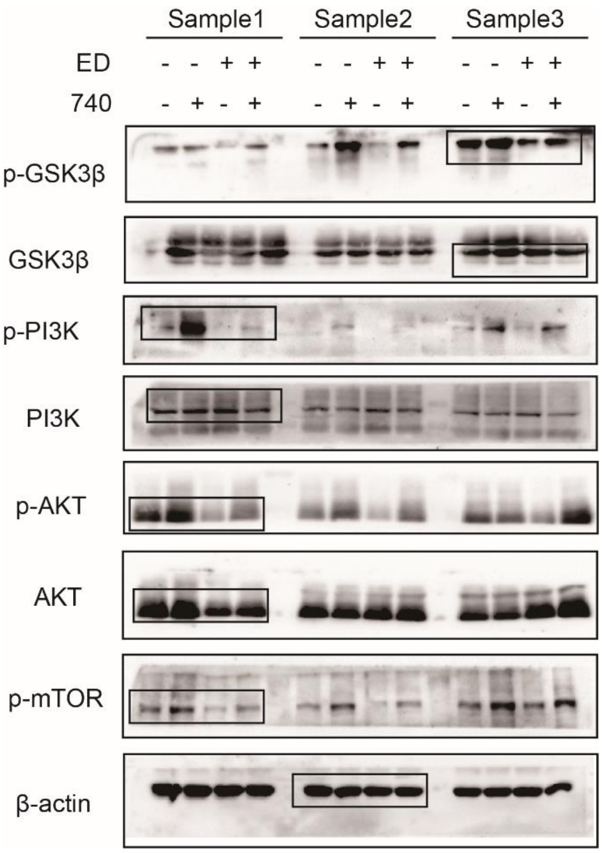

Fig. S7. The original gel pictures of Fig. 7. (A) Cells were treated with various concentrations of emodin for 48 h, and then the expression levels of p-GSK3β (Ser9), GSK3β, p-PI3K (Y607), PI3K, p-AKT (Ser473), AKT, and p-mTOR (Ser2448) were detected by western blotting. (B) Cells were pretreated with 15 μM 740 Y-P for 24 h, followed by treatment with 60 μM emodin for 48 h. The bands framed by the black line have been cropped by the original whole gel images.
