# Supplementary material for: Emodin inhibits invasion and migration of hepatocellular carcinoma cells via regulating autophagy-mediated degradation of snail and β-catenin
Source: BMC Cancer. 2022 Jun 18;22:671. doi: 10.1186/s12885-022-09684-0 (PMC9206273; doi:10.1186/s12885-022-09684-0)
Supplement: Supplementary file 4 — Additional file 4. [file 12885_2022_9684_MOESM4_ESM.pdf]

Figure S 1

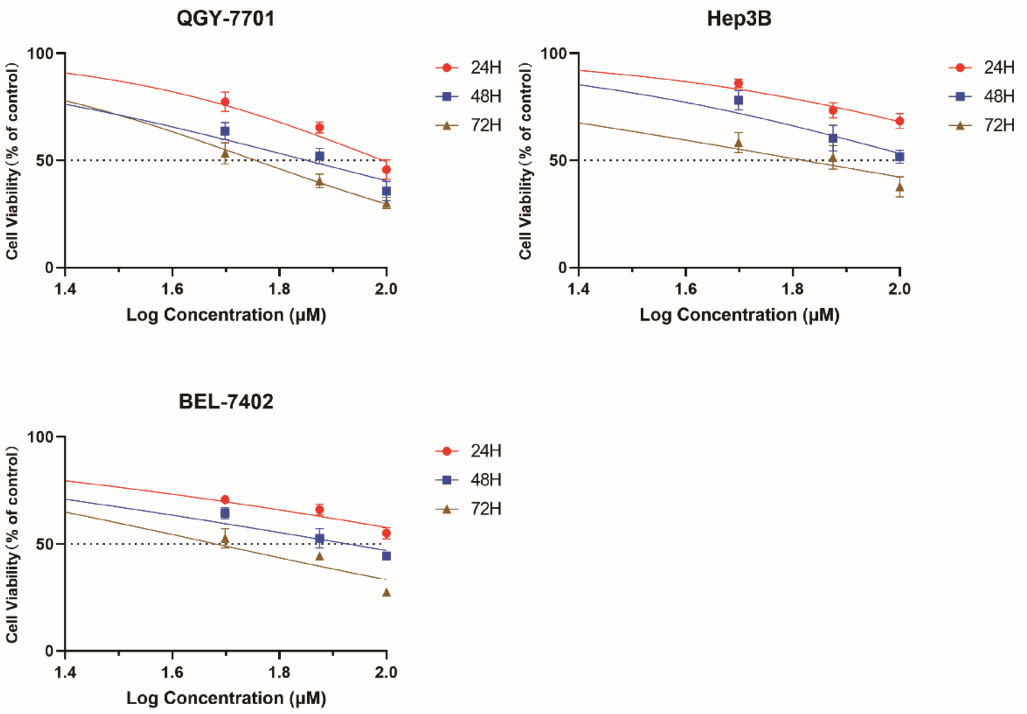

Fig. S1. Emodin inhibited cell viability in a dose- and time-dependent manner. The effect of emodin on the viability of normal liver cells and HCC was examined by MTT. At least three independent experiments were performed (n = 6).
