# Supplementary material for: Emodin inhibits invasion and migration of hepatocellular carcinoma cells via regulating autophagy-mediated degradation of snail and β-catenin
Source: BMC Cancer. 2022 Jun 18;22:671. doi: 10.1186/s12885-022-09684-0 (PMC9206273; doi:10.1186/s12885-022-09684-0)
Supplement: Supplementary file 5 — Additional file 5. [file 12885_2022_9684_MOESM5_ESM.pdf]

Figure S 2

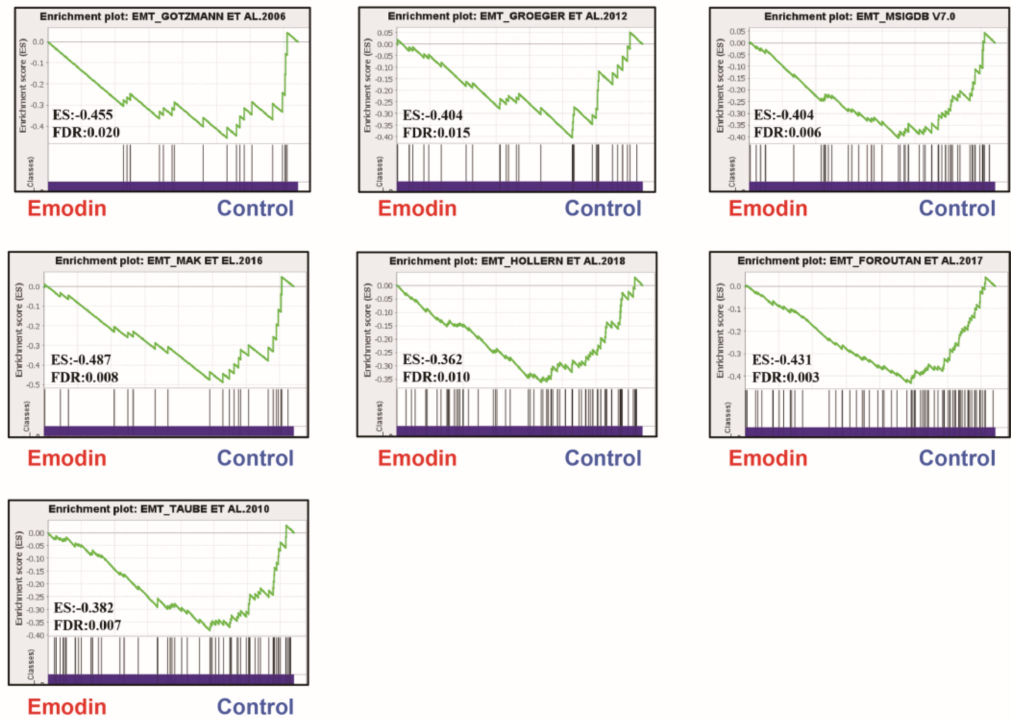

Fig. S2. Gene set enrichment analysis (GSEA) in HepG2 cells. GSEA for down-regulated genes of emodin-treated HepG2 cells in seven EMT-related gene sets.
